# Is the 360 Degree Turn Test a Safe and Reliable Tool for Post-Stroke Tele-Assessment?

**DOI:** 10.63144/ijt.2026.6730

**Published:** 2026-06-01

**Authors:** Bruna Nascimento Zanfir da Silva, Camila Pinto, Caroline Santos Figueiredo, Thainara Cruz da Rosa, Katherine Lee Hsieh, Aline Souza Pagnussat

**Affiliations:** 1 Graduate Program in Rehabilitation Sciences, Universidade Federal de Ciências da Saúde de Porto Alegre (UFCSPA), Brazil; 2 Movement Analysis and Rehabilitation Laboratory, Universidade Federal de Ciências da Saúde de Porto Alegre (UFCSPA), Brazil; 3 Graduate Program in Health Sciences, Universidade Federal de Ciências da Saúde de Porto Alegre (UFCSPA), Brazil; 4 Department of Physical Therapy, Georgia State University (GSU), Atlanta, USA; 5 Department of Physical Therapy, Universidade Federal do Rio Grande do Sul (UFRGS), Brazil

**Keywords:** Reliability, Stroke, Tele-assessment, Telehealth, Telemonitoring, Telerehabilitation

## Abstract

**Background:**

Despite the growing use of telehealth in stroke rehabilitation, it remains unclear whether online assessments can match the accuracy of in-person evaluations.

**Purpose:**

To investigate the safety and reliability of tele-assessment using the 360 Degree Turn Test in individuals with chronic stroke under controlled conditions.

**Methods:**

Twenty-five individuals with chronic stroke were assessed both in person and via synchronous tele-assessment with a 24–48-hour interval. Online assessments were conducted via video call by a trained physiotherapist. One month later, the same physiotherapist and a second physiotherapist scored the recorded assessments asynchronously.

**Results:**

There was a high level of agreement between in-person and tele-assessment (ICC > 0.90). Tele-assessment demonstrated excellent intra- and inter-rater reliability. However, participants took significantly longer to complete the test during tele-assessment (p < 0.05). No adverse events were observed.

**Conclusion:**

Tele-assessment using the 360 Degree Turn Test demonstrated high reliability and appeared to be safe under the specific conditions tested (i.e., chronic participants, caregiver presence, and controlled environment). However, performance outcomes obtained via tele-assessment may not be directly comparable to in-person assessments in longitudinal follow-up.

Stroke is a leading cause of long-term disability worldwide (Feigin et al., 2021). Several motor impairments after stroke, such as muscle weakness, increased muscle tone, and lack of coordination, may impair balance and turning movements (Lam & Luttmann, 2009; Manaf et al., 2012). For stroke patients, balance assessment is crucial for monitoring clinical progress and planning rehabilitation strategies (Shahid et al., 2023). However, traditional in-person balance assessment is not always feasible due to physical impairments, transportation barriers, or time constraints (Assis & Jesus, 2012; Shigekawa et al., 2018). Tele-assessment is an emerging approach to address these challenges and provide remote telehealth care to many patients, including those recovering from stroke (Oliveira et al., 2025; Thomas et al., 2022).

A well-established method to assess balance in post-stroke individuals is the 360 Degree Turn Test. This test has been studied as an assessment tool for community-dwelling older adults and post-stroke patients (Shiu et al., 2016). Post-stroke individuals typically require more time to turn than healthy individuals (Abdollahi et al., 2022). For post-stroke patients, the 360 Degree Turn Test has excellent reliability, with minimal detectable change values of 0.76 and 1.22 seconds for turning toward the affected and unaffected sides, respectively. The test has cutoff times of 3.49 and 3.43 seconds for turning toward the unaffected and affected sides, respectively, to distinguish between healthy older adults and stroke survivors. The 360 Degree Turn Test also correlates with other measures of physical function, such as the Fugl-Meyer assessment of the lower extremity, the Berg Balance Scale, the five times sit-to-stand test, the Timed Up and Go test, among others (Shiu et al., 2016).

Although the 360 Degree Turn Test is reliable and easily administered in post-stroke individuals (Shiu et al., 2016), the safety and reliability of its remote administration have not been fully explored. Thus, this study aimed to assess the safety and reliability of tele-assessment using the 360 Degree Turn Test in post-stroke individuals.

## Methods

### Study Design

This observational study was conducted following the Strengthening the Reporting of Observational Studies in Epidemiology (STROBE) (von Elm et al., 2008) checklist and the Guidelines for Reporting Reliability and Agreement Studies (GRRAS) (Kottner et al., 2011). Ethical approval was obtained from the Ethics and Research Committee of the Federal University of Health Sciences of Porto Alegre (Approval no.: 45137321.5.0000.5345).

The sample size was determined using WinPepi software (http://www.brixtonhealth.com/pepi4windows.html) based on a previous study (Russell et al., 2002). To ensure a minimum agreement of r=0.9, with a power of 90% and a significance level of 5%, the estimated sample size was 25 subjects. Data analysis was conducted using SPSS version 29.0 (Statistical Package for the Social Sciences, Inc., Chicago, USA).

### Subjects

Participants were recruited by convenience between December 2020 and October 2022. We included participants 18 years or older diagnosed with chronic stroke (>6 months) presenting mild, moderate, or severe hemiparesis, according to the Fugl-Meyer Assessment (Daly et al., 2011). Subjects were considered eligible based on the following inclusion criteria: no cognitive impairment (minimum score of 20 points in the Mini-Mental State Examination) (Folstein et al., 1975); access to a cell phone, tablet, or computer with a camera and internet access where evaluations could be carried out; basic knowledge of video-conferencing (participant themselves or an assistant); and a full-time caregiver or family member available during the online assessments. Eligible individuals who agreed to participate in the study signed the informed consent form.

### Procedure

Each participant underwent two random assessments comprising the same tests; one was conducted in person, and the other was conducted online. An interval of 24–48 hours was provided between the tests. The order of assessment was randomized in blocks. Both in-person and online assessments were conducted by the same physiotherapist, who visited the participants’ residences for the in-person assessment and instructed them through videoconferencing for the online assessment. A full-time caregiver or family member was required to be present during the online assessments. A guideline (OMPEPE) developed by the researchers was used to explain the execution of the online test to the participants. The guideline is presented in the Appendix.

A tutorial video was shown before each assessment, and any questions were addressed. Additionally, all tests included in the online assessment were recorded. One month later, the evaluator reviewed the tests once more to assess intra-rater reliability. A second physiotherapist reviewed and evaluated the recorded video assessment to assess interrater reliability. Safety was determined by recording any adverse events that occurred during the assessments, including falls or near-falls, cardiovascular events, and musculoskeletal injuries.

The 360 Degree Turn Test was utilized to assess the dynamic balance of the participants. First, the individual was instructed to stand with their feet comfortably apart and their arms relaxed at their sides. They were then asked to complete a full turn (360 degrees) around their axis at normal speed. The time to complete the turn and the number of steps were recorded during this process. Each participant performed three attempts in each direction (Shiu et al., 2016). The mean value of the three attempts was calculated and used for statistical analysis.

The normality of continuous variables was assessed using the Shapiro-Wilk test. Continuous variables were expressed as means and 95% confidence intervals, while categorical variables were expressed as frequency distributions. Intraclass Correlation Coefficients (ICC) were calculated using a two-way mixed-effects model with absolute agreement for single measures (ICC [3,1]), with 95% confidence intervals. The selection and reporting of the ICC model were based on established guidelines for reliability studies (Koo et al., 2016). Reliability was interpreted as follows: values <0.5 were considered poor, between 0.5 and 0.75 moderate, between 0.75 and 0.9 good, and >0.9 excellent. We compared in-person versus synchronous tele-assessment, synchronous versus asynchronous tele-assessment, and asynchronous (rater (1) versus asynchronous (rater (2) tele-assessment. The Wilcoxon test was used to compare in-person versus synchronous tele-assessment. Results were considered statistically significant if p < 0.05.

## Results

We included twenty-five individuals with post-stroke chronic hemiparesis. The characteristics of the participants are detailed in Table 1. All participants completed the entire evaluation protocol.

We found excellent reliability (ICC >0.90) for the 360 Degree Turn Test when comparing in-person and tele-assessment. However, participants took significantly longer to complete the test during the tele-assessment (p-value <0.05) (Table 2).

The 360 Degree Turn Test demonstrated excellent intra-rater reliability (when comparing online synchronous and online asynchronous). The test also presented excellent inter-rater reliability (ICC >0.90), as depicted in Table 3. This result suggests that the test can produce consistent results when administered by the same evaluator over time and when administered by different evaluators.

## Discussion

This study aimed to evaluate the reliability and safety of using tele-assessment to perform the 360 Degree Turn Test in individuals post-stroke. The study findings revealed that tele-assessment using the 360 Degree Turn Test yielded highly consistent results, as evidenced by the agreement between in-person and online assessments and the excellent intra-rater and inter-rater reliability scores. Additionally, no adverse events were reported, even in a sample including individuals with moderate to severe impairment. These findings suggest that the 360 Degree Turn Test conducted through tele-assessment is safe under the specific conditions of this study, including chronic post-stroke participants, the presence of a caregiver or family member, and a controlled testing environment.

Notably, this agreement refers to the degree of consistency between the two forms of test delivery and between two evaluators assessing the same patient. However, it does not mean that the results obtained by in-person and tele-assessment are identical. In the present study, participants took longer to complete the test during the tele-assessment. Considering the minimal detectable change values of 0.76 and 1.22 seconds for turning toward the affected and unaffected sides, respectively (Shiu et al., 2016), the observed differences between the two administration methods (1.17 for the affected side and 0.77 for the unaffected side) were both statistically and clinically significant for the affected side. Therefore, these findings suggest that in-person and tele-assessments may not be directly comparable in clinical practice.

Tele-assessment using the 360 Degree Turn Test demonstrated high reliability and appeared to be safe under the specific conditions of this study. While it is noteworthy that the interrater reliability scores were excellent, indicating consistent measurements of the same phenomenon, it is important to consider the potential for bias as the raters used different assessment formats (online asynchronous vs. online synchronous). Furthermore, although the use of the same physiotherapist is consistent with intra-rater reliability assessment, it may also have contributed to increased agreement, as prior exposure to the participants’ performance could have influenced scoring during video reassessment. This should be considered when interpreting the high reliability values observed.

Additionally, it is important to note that participants may take longer to complete the test during tele-assessment. As assessments were conducted in the same environment, these differences are unlikely to be explained by environmental variability. Instead, they may reflect behavioral and contextual factors inherent to remote assessment, such as increased caution due to the absence of the examiner’s physical presence, reduced confidence during movements not physically supervised, or subtle delays related to verbal instruction and feedback via videoconferencing. These factors should be considered when interpreting results obtained through tele-assessment. Additionally, a mixed evaluation approach may introduce variability that should be considered when interpreting results. Finally, the use of a standardized guideline during test administration may have contributed to the high level of agreement observed. Overall, these findings highlight both the potential and the limitations of tele-assessment for post-stroke balance evaluation.

## Figures and Tables

**Table 1:** Sample’s Characteristics

Variables	Individuals post-stroke (n=25)

Age (years)	60.9 (55.70–66.13)

Sex (Male/Female)	18/7

Educational level	
Basic education	5
High school	4
Higher Education	16

Time since stroke (months)	55 (35.51–74.48)

Stroke type (Ischemic/Hemorrhagic)	17/8

Affected side (Right/Left)	8/17

Mini-Mental State Examination	27.3 (26.12–28.43)

FMA-LL (Severe/Moderate)	13/12

*Note*. Data are expressed as frequencies, means, and standard deviations. FMA-LL: Fugl-Meyer Assessment - lower limb; n: number of participants.

**Table 2:** In-person Versus Tele-assessment

Assessment	In-person	On-line synchronous	P-value	ICC (95% CI)
360TURN AS (s)	12.74 (9.06–16.42)	13.91 (9.89–17.93)	0.016*	0.98 (0.94–0.99)
360TURN AS (steps)	11.71 (9.71–13.71)	12.30 (10.08–14.52)	0.094	0.97 (0.93–0.98)
360TURN US (s)	13.48 (9.36–17.61)	14.25 (10.03–18.47)	0.032*	0.98 (0.96–0.99)
360TURN US (steps)	12.84 (10.31–15.37)	13.06 (10.53–15.59)	0.75	0.98 (0.95–0.99)

*Note*. AS: affected side; US: unaffected side; 360TURN: 360 Degree Turn Test; R: right; L: left; s: seconds; ICC: Intraclass Correlation Coefficient; CI: confidence interval. Data are mean and confidence intervals.

**Table 3:** Intraclass Correlation Coefficient (ICC) for Inter-rater and Intra-rater Reliability

Assessment	Evaluator 1 (on-line synchronous)	Evaluator 1 (on-line asynchronous)	ICC (95% CI)	Evaluator 2 (on-line asynchronous)	ICC (95% CI)
360TURN AS (s)	13.91 (9.89–17.93)	13.73 (9.83–17.62)	0.99 (0.99–0.99) a	13.87 (9.96–17.78)	0.99 (0.99–0.99) b
360TURN AS (steps)	12.30 (10.08–14.52)	12.29 (10.05–14.53)	0.99 (0.99–1.00) a	12.28 (10.06–14.51)	0.99 (0.99–1.00) b
360TURN US (s)	14.25 (10.03–18.47)	14.33 (10.08–18.59)	0.99 (0.99–1.00) a	14.52 (10.19–18.85)	0.99 (0.99–0.99) b
360TURN US (steps)	13.06 (10.53–15.59)	13.06 (10.50–15.63)	1.00 (0.99–1.00) a	13.07 (10.48–15.62)	0.99 (0.99–0.99) b

*Note*. AS: affected side; US: unaffected side; 360TURN: 360 Degree Turn Test; R: right; L: left; s: seconds; ICC: Intraclass Correlation Coefficient; CI: confidence interval;

a – intra-rater reliability;

b – inter-rater reliability.

Data are mean and confidence intervals.

## Appendix

### OMPEPE Guideline for 360 Degree Turn Test

**Table:**

*360 Degree Turn Test*	
**O**	We will administer this test, which is called the 360 Degree Turn Test, to evaluate your dynamic balance, which is your ability to maintain balance while moving.
**M**	This test does not require any materials.
**P**	To begin the test, you must be standing facing me with your legs comfortably apart and your arms relaxed.
**E**	When I give you the command, make a complete 360-degree turn around your own axis.
**P**	At the end of the test, your position should be facing me again.
**E**	The testing area must be clear of any obstacles or objects that could disrupt the test or draw your attention away.
Observation: We will perform the test three times for each side to ensure accuracy. You can use devices to aid during the test, such as crutches if necessary.	
